# Items

**Published:** 1882-03

**Authors:** 


### Items.

THE *College and Clinical Record*, one of our most esteemed exchanges—taking as a text a complimentary allusion, made by the REGISTER, to an article recently published in its columns—proceeds to define its position, and to explain that the *Record* was instituted in the interests of the alumni and students of the Jefferson College by two graduates of that institution. It claims to be entirely independent of the trustees and faculty. It is in no sense the organ of the school, and does not represent the faculty in any way, nor has it ever received from that body any assistance of any kind whatever. The *Record* deserves the support of not only the alumni of the Jefferson College, but also of the entire medical profession. It is entitled to the success which it has achieved, and we heartily wish it a long and prosperous career.

---

THE COLLEGES.—The lecture session is over in both of the colleges of this city. On Monday 27th inst., the

Atlanta Medical College holds its annual commencement at DeGive's Opera House. The somewhat famous politician, Dr. W. H. Felton, makes the address. Mr. J. C. Avery is the class valedictorian.

Two days later, March 1st, the Southern Medical College holds its annual commencement exercises. Rev. A. J. Battle, D.D., President of Mercer University, makes the annual address. Mr. J. H. Sims, of Newton county, is class valedictorian.

We learn one of the eclectic colleges has its annual exercises in the same hall during the latter part of the week. Verily, the doctors hold the boards for that week.

---

WE print, in the appropriate section, an abstract of the proceedings of the Atlanta Medical Society. We shall strive to publish these reports regularly, and we feel confident that they will constitute an interesting, instructive and popular feature of the REGISTER. It has been, and it shall continue to be the aim of this Journal to develop, in every possible way, the resources and the influence, and to promote the real good of the medical profession. Everything which encourages organization and community of effort tends in this direction, hence all properly conducted medical societies will receive our cordial support and encouragement.

---

STRANGE BUT TRUE.—At one of the public vaccination offices in this city, recently, the gentleman in charge, after having vaccinated a little girl, requested her mother to return with her on the eighth day, as he might then desire to use some lymph from her arm. The mother demurred, and explained to the Doctor, in the most confiding manner, that the child had been bitten by a dog in infancy, and that she was afraid the virus would transmit hydrophobia.

Thus another argument is unwittingly supplied to the anti-vaccinationists and to the "bovine men."

---

THE *Southern Dental Journal* makes its debut with the February number. It is edited by B. H. Catching, D.D.S., and published monthly, in Atlanta, by R. A. Holliday.

The initial number presents an attractive appearance; the original and selected articles are well chosen, and it gives promise of being a live and useful periodical. We trust the enterprise will receive the support which it merits, and that it may reap a large measure of well earned success.

---

NEW PREPARATIONS.—It is getting time that the profession set its face against the avalanche of new remedies (at least, claimed as such) and the myriads of new and fancy preparations constantly thrust under the professional nose by ambitious manufacturers. In the main, there is no good in them except for the manufacturers; and time will prove there is not much in it for the majority of that class.

---

THE *Alienist and Neurologist*, bestows high praise upon Dr. Robt. Battey, of this State, formerly editor of this Journal (old series), for his remarks at the International Congress, upon the operation which has made him so famous. The conservatism of the originator, as compared with the reckless zeal of some of his followers, is especially dwelt upon.

---

IT is said that Dr. J. W. Draper, who died January 4th, left a fortune of one hundred thousand dollars of his own making. He was an eminent scientist, teacher, and author. Among his works are The Conflict Between Science and Religion, and a history of our late civil war.

---

HOT AND COLD APPLICATIONS.—The Cincinnati Academy of Medicine has been engaged in discussing the relative merits of each in inflammation and other affections. This question has always been a little mixed in the minds of some of the profession.

---

SAMPLE COPY FIEND.—Some of the exchanges seem to be annoyed by him. The plain business proposition is, if you don't want to send a copy, don't do it. The law does not compel you.

VIVISECTION.—Dr. Ferrier, of London, who was prosecuted by the Anti-Vivisection Society, for violation of some provision of an act governing said practice, was not convicted; but merely because they had caught the wrong man by the ear.

---

THE *Louisville Medical New* comes to this office addressed to the *ECLECTIC* MEDICAL REGISTER. What we have done to merit this title of distinction we know not; but the fact remains.

---

THE new college established in Chicago claims that the "examinations for a degree will be conducted by a committee of examiners separate and distinct from its teaching corps."

---

BOGUS DIPLOMAS.—It is said that Buchanan, who formerly ground out diplomas in Philadelphia, is about to, or has already made an attempt to establish a similar mill in Detroit.

---

PROOF FOR HOMŒOPATHY.—We recently heard a lady offer as proof of *similia similibus curantur*, that vaccination (which she thought was from small-pox) prevented that disease.

---

WE have heard that a prominent physician of this city discountenances vaccination. He should be put in a glass case in a museum of Egyptian curiosities.

---

SEVERAL writers and correspondents are taking Keith to task for abandoning Listerism.

---

PHARMACY.—A new "pharmacy bill" is before the Kentucky legislature.

---

IT takes one hundred thousand dollars a year to support Bellevue Hospital.

